# Pathophysiology, risk, diagnosis, and management of venous thrombosis in space: where are we now?

**DOI:** 10.1038/s41526-023-00260-9

**Published:** 2023-02-16

**Authors:** Katie M. Harris, Roopen Arya, Antoine Elias, Tobias Weber, David A. Green, Danielle K. Greaves, Lonnie G. Petersen, Lara Roberts, Tovy Haber Kamine, Lucia Mazzolai, Andrej Bergauer, David S. Kim, Rik H. Olde Engberink, Peter zu Eulenberg, Bruno Grassi, Lucrezia Zuccarelli, Giovanni Baldassarre, Kevin Tabury, Sarah Baatout, Jens Jordan, Andrew P. Blaber, Alexander Choukér, Thais Russomano, Nandu Goswami

**Affiliations:** 1 Faculty of Medicine, Memorial University of Newfoundland, St. John’s, Canada; 2 Kings College Hospital, NHS Foundation Trust, London, UK; 3 Vascular Medicine, Toulon Hospital Centre, Toulon, France; 4 Space Medicine Team, European Astronaut Centre, European Space Agency, Cologne, Germany; 5 KBR, Cologne, Germany; 6 Centre for Human and Applied Physiological Sciences, King’s College London, London, UK; 7 Faculty of Health, University of Waterloo, Waterloo, Canada; 8 Department of Biomedical Sciences, University of Copenhagen, Copenhagen, Denmark; 9 Department of Aeronautics and Astronautics, Massachusetts Institute of Technology, Cambridge, USA; 10 Division of Trauma, Acute Care Surgery, and Surgical Critical Care, Baystate Medical Center, Springfield, MA USA; 11 Department of Angiology, Lausanne University, Lausanne, Switzerland; 12 Surgery, LKH Südsteiermark, Wagna, Austria; 13 Department Emergency Medicine, University British Columbia, Vancouver, Canada; 14 Amsterdam UMC location University of Amsterdam, Department of Internal Medicine, Section of Nephrology, Amsterdam, The Netherlands; 15 Amsterdam Cardiovascular Sciences, Microcirculation, Amsterdam, The Netherlands; 16 Institute for Neuroradiology, Ludwig-Maximilians-University Munich, Munich, Germany; 17 Department of Medicine, University of Udine, Udine, Italy; 18 Radiobiology Unit, Belgian Nuclear Research Centre, SCK CEN, Mol, Belgium; 19 Institute of Aerospace Medicine, German Aerospace Center and University of Cologne, Köln, Germany; 20 Department of Biomedical Physiology and Kinesiology, Simon Fraser University, Burnaby, BC Canada; 21 Translational Research Stress & Immunity, Klinik für Anästhesiologie/Forschungslabors, LMU Klinikum, München, Germany; 22 InnovaSpace UK, London, UK; 23 Division of Physiology, Otto Löwi Research Center for Vascular Biology, Immunity and Inflammation, Medical University of Graz, Graz, Austria; 24 Mohammed Bin Rashid University of Medicine and Applied Health Sciences, Dubai, United Arab Emirates

**Keywords:** Risk factors, Diagnostic markers, Thrombosis

## Abstract

The recent incidental discovery of an asymptomatic venous thrombosis (VT) in the internal jugular vein of an astronaut on the International Space Station prompted a necessary, immediate response from the space medicine community. The European Space Agency formed a topical team to review the pathophysiology, risk and clinical presentation of venous thrombosis and the evaluation of its prevention, diagnosis, mitigation, and management strategies in spaceflight. In this article, we discuss the findings of the ESA VT Topical Team over its 2-year term, report the key gaps as we see them in the above areas which are hindering understanding VT in space. We provide research recommendations in a stepwise manner that build upon existing resources, and highlight the initial steps required to enable further evaluation of this newly identified pertinent medical risk.

## Introduction

A recent study investigating astronaut jugular venous flow inflight on the International Space Station (ISS) reported the incidental finding of a persistent asymptomatic obstructive left internal jugular (IJV) venous thrombosis (VT) in a single crewmember^1^. Furthermore, an additional suspected case was reported following retrospective analysis of the images from the other 11 crew members who participated in the study^2^. Venous thrombi can lead to localised pain, redness, distal swelling, venous ischaemia, skin necrosis and even organ dysfunction^3^. However, the most significant complication is lung embolization of thrombotic fragments, termed pulmonary embolism (PE) that can lead to cardiorespiratory insufficiency, which can be fatal. Thus, the potential identification of VT, a mission-critical medical condition, in such a highly selected, previously considered low-risk population is concerning and warrants urgent investigation.

As a result, the European Space Agency (ESA) supported the creation in 2020 of a ‘Topical Team’ comprising of international subject matter experts in coagulation, venous thrombosis, prophylaxis, and treatment of thrombo-embolic events. The creation of this team was additionally supported by the Cardiovascular, Pulmonary, Renal and Lymphatic Working Group of the ESA SciSpacE roadmap. An operationally focused research strategy is required to define the pathophysiological processes leading to VT presentation in spaceflight, potential clinical presentation, its risk (and the factors that may affect risk), whilst proposing evaluation of candidate diagnostic, mitigation, and management strategies compatible with human spaceflight. The team was supported by individuals familiar with Human Spaceflight medical operations to identify relevant knowledge gaps.

The objectives of the team were to:

1. Identify key knowledge gaps and technological/procedural development needs in the (proposed) domains of:
  11. Venous system status, hemodynamics and coagulation cascade in microgravity (Pathophysiology working group);
  12. Risk of VT and its clinical presentation in microgravity (Risk working group);
  13. VT diagnostic systems compatible with the constraints of spaceflight (Operational Diagnostic working group);
  14. Management of VT in microgravity (Operational Management working group).
2. To determine a cohesive and appropriate operationally driven research and technological/procedural evaluation strategy that addresses the identified gaps, needs, risks and risk factors, whilst also proposing evaluation pathways of candidate diagnostic, mitigation and management strategies compatible with future long duration/exploration space missions.

Initially four working groups were formed to address the specific domains, however during monthly full group meetings where ongoing research and key findings of the subgroups were discussed, working groups I & II were combined. This paper will discuss the findings of the topical team, key knowledge gaps, and provide recommendations for future research along European and international space research goals.

## Venous thrombus in space: what do we know?

### VT Pathophysiology and risk factors (working group I)

To date, no symptomatic cases of VT have been identified in 609 astronauts that have undertaken space travel (https://www.worldspaceflight.com/bios/stats.php). In 2019, a cohort study of 11 ISS crew members showed 6 individuals with apparent stagnant or retrograde IJV flow on approximately flight day 50, with one developing occlusive IJV thrombosis^2^, the management of which is discussed separately by Aunon-Chancellor et al^1^. An additional potential partial IJV thrombus was identified in another crew member retrospectively^1,2^.

These findings suggest that the underlying VT incidence in space may not be dissimilar to that in the general population. For instance, the average rate of venous thrombus in the United States is 1–2 per 1000 people^4^. A calculation of the 95%CI of the proportion states that a symptomatic VT rate of 0/609 astronauts places the upper bound of the 95%CI at 0.6%, similar to the symptomatic VT rate in postoperative patients (at 0.3–0.7%)^5^. Concerningly, VT in neck veins may be associated with relatively poor outcomes^6^, yet are rare on Earth in the absence of specific risk factors^7^, accounting for 4–10% of overall VT or ~4–10 per 100,000 persons per year^8^. Currently, astronauts are a carefully selected cohort that lack many classical VT risk factors (detailed in Table 1), however, studies suggest that up to 50% of VT on Earth have no identifiable risk factors^9^.

**Table 1:** VT risk factors on Earth. Categorized as strong (OR > = 10); moderate (OR 2–9) or weak (OR < 2) Adapted from Table 3, Ortel et al.^28^.

Weak risk factors	Moderate risk factors	Strong risk factors
Bed rest/immobility	Combined hormonal contraception/hormone replacement therapy	Prior VT
Age	Inflammatory/autoimmune diseases	Recent major surgery/major trauma
Varicose veins/venous insufficiency	Infections	Antiphospholipid syndrome/ Antithrombin deficiency
Superficial vein thrombosis	Congestive heart or respiratory failure	Active cancer^15^
Obesity	Venous catheters	Recent hospitalisation or more than a 3-day immobilisation for acute medical conditions
	Pregnancy or postpartum period	Neurological disease with extremity paresis^9^
		Genetic thrombophilia

There may be space- or microgravity-specific risk factors, such as the removal of tissue compressive forces along with cephalad fluid shifting that may also cause pathologies such as Spaceflight Associated Neuro-ocular Syndrome (SANS)^10,11^, which is suggested by the finding of flow abnormalities in the aforementioned studies. However, whether IJV flow was correctly identified as stagnant or retrograde in the initial cohort study, considering the technological limitations and the relative inexperience of crew as sonographers, is unknown^12^. However, a recent update by Pavela et al. ^13^ also documented retrograde IJV flow in 2/11 astronauts undergoing routine venous ultrasound surveillance in flight.

A recent systematic review reported that microgravity and its ground-based analogues, chiefly 6° head down tilted bed rest (HDTBR), may induce an enhanced coagulation state, most prominently in the venous system due to cephalad fluid shifting during gravitational unloading^14^. Some evidence of changes in venous flow, distension, pressures, endothelial damage, and possibly hypercoagulability exists, suggesting that all aspects of Virchow’s Triad may be modulated but the data is not conclusive^14^. While unexplored by Kim et al. ^14^, the radiation environment in Low Earth Orbit (LEO) may also have an effect, particularly as radiotherapy is associated with a significant increase in the risk of VT, including cerebral bleeding^15^. However, whether such changes precipitate an actual increased VT risk in spaceflight remains to be determined, given the lack of VT in ground-based analogues and limited sample of VT in spaceflight. Additionally, the association of radiotherapy with VT on the ground is often within the context of malignancy, which may be a risk.

Following spaceflight, a return to activity in a 1 G gravitational field may also constitute a VT risk factor. Indeed, changes in G-loading have been demonstrated to be high risk periods, with brief periods of hypergravity activating haemostasis in healthy volunteers^16^. However, the only documented VT to date in spaceflight was undetectable upon landing, and was discovered months after the hypergravity of launch^1^. While a return to 1 G after prolonged exposure to 0 G may induce haemostasis due to the relative hypergravity of the changed gravitational field, this has not been thoroughly explored.

One set of VT risk factors of increasing relevance are those related to sex^17,18^ given that whilst to date (as of March 2022), only 67 women have flown into space^19^, representing <10% of the astronaut population^20^. This situation is evolving, with 50% (9/18) of those selected by NASA in 2020 to prepare for the Artemis programme (NASA Artemis. at https://www.nasa.gov/specials/artemis/) being females. Indeed, whilst there are some physiological differences that should be considered^21^, females may also present operational physiological advantages for extended spaceflight due to decreased (on average) body size and energy consumption^22^ compared to their male counterparts^23^. Sex-based risks include the optional but frequent use of hormonal contraception in female astronauts to suppress menstruation^17,18,24,25^. The elevated risk is highest during the first months but remains significantly increased compared to non-users, depending on the type of hormonal contraceptive prescribed (ie. 1st or 2nd generation, (2–3x risk), versus 3rd or 4th generation (2–5x risk))^18,26,27^. Moreover, increased risk persists up to 3 months following discontinuation^18^. The presence of thrombophilia further increases VT risk. There is currently no indication for hereditary or acquired thrombophilia screening in astronauts without a personal or family history of VT or without any other comorbidity. Whether this needs to be re-considered remains to be determined—particularly as thrombophilia testing has little relevance for terrestrial VT management^28,29^.

Finally, as space travel is opening to a wider population, conventional risk factors associated with VTs may become more relevant to the practice of aerospace medicine. While the medical requirements for private spaceflight participants are currently less stringent than space agency crews, they will evolve^30^. Consideration of pre-existing conditions and comorbidities such as age, obesity, personal history of VT (VT or PE), family history of unprovoked VT and cancer (and its treatment) will become even more important for longer duration spaceflight participants.

### VT diagnosis

Given the limited resources^31^ and absence of clinical presentation, VT diagnostic testing is not performed in space. However, in symptomatic patients a range of approaches are taken.

On Earth, B-mode compression ultrasound (CUS) is reported to be a sensitive and specific diagnostic method for acute VT in the peripheral veins of both the lower and upper limbs in addition to the jugular veins^32–35^. The technique relies on venous compressibility to ‘rule-out’ the presence of VT, with non-compressibility combined with the direct imaging of the thrombus confirming a VT diagnosis. Colour Doppler Ultrasound is an adjunct to B-mode CUS. By evaluating venous blood flow, it helps to delineate the thrombus if it is partially obstructing the vein, or show no flow when the thrombus is completely occlusive^32,34,35^.

Slow, stagnant, or retrograde venous flow may be important clues to understanding if a clot is formed, in the process of forming or even predict where one may in the future. The combination of Colour Doppler Ultrasound for the direct investigation of central vein thromboses (abdominal, pelvic and intrathoracic veins) with the Doppler signal patterns at the level of the peripheral veins appears to provide high diagnostic accuracy, although this technique has not been subject to the same level of validation compared to peripheral veins in the legs^32,33^.

In spaceflight, the diagnostic accuracy of conventional B-mode US/ CUS/ Colour Doppler US/ Doppler US is unknown. Furthermore, reduced, stagnant and/or retrograde venous flow may occur not due to VT, but rather—particularly in the upper-limb and jugular veins—due to volume overload and elevated venous pressures secondary to the classic cephalad fluid shift^36^. In fact, the ultrasound used to measure venous blood flow velocity on ISS, and subsequently qualitatively to determine the presence of turbulent, slow, stagnant or retrograde venous flow is intended to be applied to the high velocities associated with arterial flow^12^. That said, discerning the presence or absence of flow is sufficient in most cases, if the pressure applied at the probe is appropriate and consistent.

Hence, whilst the compression ultrasound (CUS) test can rule-out the diagnosis of thrombosis in a peripheral vein if it is compressible, it may lack specificity if the vein segment is not compressible in spaceflight. This lack of specificity due to higher pressures needed to compress, and thus collapse a vein may potentially be alleviated by simply using an adjacent artery as a visual ‘pressure gauge’. For instance, by increasing the pressure applied at the ultrasound probe to that required to almost close the adjacent artery, the visualised vein segment should be completely compressed^37^.

It must be noted however that manual control of applied pressure in microgravity is difficult even if combined with the use of the Crew Medical Restraint System (CMRS). Currently, safety concerns relating to pressure control preclude the use of autonomous probes. Thus, even with remote guidance (from ground-based sonographers), the crew, despite being inexperienced sonographers, must generate application pressure and thus significantly affect image acquisition. This is particularly important in, and around the neck as inadvertent activation of the carotid sinus baroreceptors could precipitate hypotension and bradycardia in individuals with carotid sinus hypersensitivity^38^. Thus, poor technique and image quality could translate into flow measurement error, particularly in the case of no-flow characterisation.

Furthermore, ultrasonic flow evaluation requires several assumptions including linear flow with respect to the vessel lumen and probe positioning^12^. This, assumption is in fact known to be violated in the IJV^39^. However, angle-independent methods are being developed in addition to technologies such as ultra-fast US and vector projectile imaging that show promise in facilitating more accurate evaluation of turbulent, slow, stagnant, and retrograde flow^40^.

D-dimer (product of fibrin, a protein fragment present in blood following fibrinolytic clot degradation) is a test used terrestrially to rule out lower limb VT and PE^3^ although it is less established for the diagnosis of upper-limb VT. The key utility is that a low D-dimer in conjunction with a low Wells score can be used to exclude lower limb VT/PE without further imaging^41^. However, given the lack of clarity regarding sensitivity to VT in the upper body and the ‘normal range’ in spaceflight it is unclear whether D-dimer assessment would be a valid diagnostic approach. Additionally, D-dimer is not currently available or validated for the spaceflight context, nor is the Wells score, and therefore is of limited interest for future diagnosis of VT during spaceflight.

### Management

Thus far, the experience in managing IJV thrombosis in space is limited to the single asymptomatic individual. The astronaut was anticoagulated with once daily enoxaparin (1.5 mg/kg, then 1 mg/kg to extend supplies) transitioning to apixaban 5 mg twice daily at 42 days, with dose reduction at 3 months. To negate the risk of excessive bleeding due to traumatic injury on re-entry treatment, the anticoagulant was stopped 4 days prior to return to Earth^1^. Thought was also given to the provision of an antidote in the event of anticoagulation-related bleeding^1^.

On Earth, oral anticoagulants including direct factor Xa inhibitors (rivaroxaban, apixaban, edoxaban), direct thrombin inhibitors (dabigatran), vitamin K antagonists (warfarin), and subcutaneous anticoagulants such as low molecular weight heparins or fondaparinux are approved for VT management. Current international guidelines or consensus documents^42^ recommend direct oral anticoagulants (DOACs) as the first line treatment in the absence of contraindications.

Furthermore, antidotes are available for rivaroxaban and apixaban (andexanet alfa), dabigatran (idarucizumab) and warfarin (vitamin K and prothrombin complex concentrate)^43^ but not for LMWH, though there is partial efficacy of protamine for this purpose^44^. The availability of an antidote is a key advantage as rapid reversibility may be required in the case of traumatic injury and may reduce the need for monitoring. However, costs in replacing medications with limited shelf life may be prohibitive and impractical in the face of the low likelihood of VT and subsequent anticoagulant-related bleeding.

On Earth, anticoagulation medication is usually prescribed for 3 months in patients with proximal VT if the risk of recurrence is low (i.e., major transient/reversible risk factors)^28^. Longer term anticoagulation medication can be considered for those with low bleeding risk and high recurrence rate (i.e., major thrombophilia, persistent or non-identified risk factors). A further important consideration is the risk of embolization from VT in spaceflight. On Earth, upper extremity VT is associated with a significantly (5–8%) lower embolization risk than lower extremity VT^45^. However, theoretically, IJV VTs could propagate to more proximal vein segments and cause superior vena cava thrombosis and cannot be simply extrapolated from Earth to space.

## Gap analysis and recommendations

Over the course of the topical team’s tenure, we have published on the likelihood of flow abnormalities in contributing to VT formation^12^, endothelial disruption related risks of VT^46^, and diagnostic modalities for VT in space (in review). We found that current tools on board the ISS are insufficient to characterize blood flow patterns that may contribute to clot formation and that there are no validated biomarkers that reliably predict increased likelihood of VT formation in otherwise healthy people. Based upon these publications and through the meetings of the topical team, knowledge gaps in each of the working group areas were identified. These key knowledge gaps are shown in Tables 2–5, along with recommendations for future research to address said gaps.

**Table 2:** Pathophysiology/Risk—Gap Analysis and Recommendations.

Gap	Recommendation
We do not know the specific VT risk associated with spaceflight, or the modulating factors of spaceflight that affect risk (ie. length of flight).	Establish the relative risk profile of VT during spaceflight, together with anatomical predilection sites (due to headward fluid shift and other changes). A comprehensive investigation of the whole venous network (peripheral and central vein segments) is essential to clearly distinguish between thrombosis and stasis in spaceflight and to identify potential sites of thrombosis and key areas to screen during future flights.
We do not know the utility of screening potential astronauts for thrombophilia’s or terrestrial VT risk factors.	Evaluate the use of test specifically aimed at evaluating endothelial/microvascular function (e.g., the increase in blood flow in the common femoral artery, determined by Eco-Doppler, during passive leg movements) in the selection of astronauts. Consider routine screening for VT in future travellers. Significant effort must be made to develop state-of-the-art technologies to individualize direct imaging of the thrombus at the level of the peripheral veins (lower and upper limbs) and the central veins (abdomen, pelvis, and thorax). The group recommends that ESA creates a Request for Proposal to further investigate high frame rate ultrasound and 3D motorized probes as next generation diagnostic technology for quantifying blood flow characteristics and possibly predicting a circulation environment where clots might occur. Consider the feasibility of spaceflight for individuals with specific known risk factors (i.e., Factor C/S deficiency, genetic risks).
We do not know the risks or benefits of hormone therapy in female astronauts.	Further assess potential prothrombotic effects of sex hormones during space travel.
We do not know the pathophysiological processes that may underlie symptomatic and asymptomatic VT risk.	Further assess microgravity-specific potential risk factors. Build a database of standard measures (biomarkers and ultrasound flow) to track changes and timeline of changes during analogue trials and in-flight missions.
We do not know the normal range of values in- and immediately post-flight of candidate Earth-based coagulation tests.	Assess prothrombotic biomarkers during spaceflight and in ground-based analogues. Spaceflight testing of coagulation markers and coagulation times is necessary to recommend treatment protocols.

**Table 3:** Prevention—Gap Analysis and Recommendations.

Gap	Recommendation
We do not know which measures could be taken to prevent VT in space. We do not know the adverse effects of potential countermeasures.	1. Assess the role of countermeasures and potential adverse effects of those countermeasures for prevention of VT, including: a. LBNP in VT prevention during space travel and in ground-based analogues^36^ b. Assess effectiveness of whole-body vibration (WBV) and RVE interventions to prevent VT during spaceflight and in ground-based analogues c. Assess role of anticoagulant and antiplatelet therapy for VT prevention during spaceflight and in ground-based analogues d. Assess the role of nitric oxide (NO) precursors (nitrites and nitrates) as a way to increase NO bioavailability, induce vasodilation, and prevent inappropriate aggregation / coagulation. e. Assess role of mechanical measures (e.g., compression stockings) to reduce stasis during spaceflight and in ground-based analogues f. Assess the role of exercise (type of exercise, intensity, duration, time of the day) as a way to increase prevent muscle atrophy (and the associated decreased muscle pump), induce shear stress, improve endothelial function, prevent venous stasis, inappropriate aggregation and coagulation.

**Table 4:** Diagnosis - Gap Analysis and Recommendations.

Gap	Recommendation
We do not understand the effects of microgravity on conventional coagulation/clotting tests.	Further assess coagulation parameters in microgravity and their role in diagnosing VT.
We do not have any means of measuring coagulation activity in spaceflight to date, nor have we identified alternative devices that could be useful during spaceflight to assess coagulation function.	Determine how microgravity affects the validity/standards of the tests of coagulation systems and function. Investigate the potential utility of viscoelastic tests such as TEG, TEM, or Sonoclot, which are cartridge based and easy to use, and capable of assessing the entirety of the clotting cascade. Baseline values of TEG/TEM in spaceflight participants would be required.
We do not have a valid spaceflight compatible imaging diagnosis pathway.	Determination of a gold-standard diagnostic algorithm/pathway (biomarkers and imaging). This involves progressing existing work on diagnostic methods (U/S and biomarkers) for VT during spaceflight^46^. The ultrasound criteria to rule-out and rule-in the diagnosis of VT in spaceflight must be established. The group recommends that ESA directs its resources to procuring the highest-quality and cutting-edge technology in ultrasound devices for use on Artemis and longer duration missions. Create a diagnostic flowchart based on pre-test and post-test probability. Progress existing ultrasound technologies available to crew for diagnosis and prediction of VT based on turbulent flow patterns or changes to flow patterns with fluid shifts.

**Table 5:** Management—Gap Analysis and Recommendations.

Gap	Recommendation
We do not know potency or efficacy of traditional anticoagulant therapies in the microgravity environment.	The risks of anticoagulation in space and optimal reversal strategies for anticoagulants during space flight must be determined, specifically the role of DOACs and their reversal agents. Characterize the anticoagulant drug levels and pharmacokinetics we can expect in space travellers and the haemodynamic effects of anticoagulants in microgravity.
We do not know if follow-up from a VT event should be conducted in space or on the ground post mission.	Access existing longitudinal health data from astronauts to assess long term cardiovascular health including sequelae of VT to determine if existing long term follow up is suitable.

**Fig. 1:**
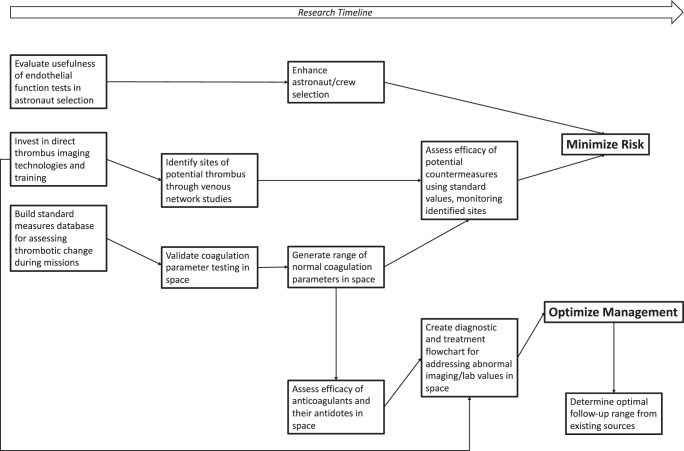
Suggested areas of research based on ESA Topical Team gap analysis. Each box contains a research focus that will build towards better characterization of risk and/or management. The dependencies between the different research foci are shown by the arrows.

While the topical team was subdivided into four initial subgroups, the main objective of the team, and space medicine, is to prevent medical emergencies from occurring, and developing a reasonable management plan for the rare instances they do occur. To capture this goal and show the interdependencies of our findings, a visual representation of the above recommendations and the pathways in which they enable the primary prevention and management of VT in space is shown in Fig. 1, considering the recommendations which must be addressed first to enable the implementation of the other recommendations.

## Discussion

There continue to be many unknowns regarding the pathophysiology, risk, prevention, diagnosis, management, and follow up of VT occurrences during spaceflight. With upcoming missions to the Moon and Mars, differences in medical resources and crew autonomy will further complicate the priority of each of the above recommendations. For example, advanced diagnostics that require from-ground input are unlikely to be undertaken for missions with significant communication delays, while initial investigation into the nature of clotting in microgravity may be undertaken preferentially in the low Earth orbit environment. The VT Topical Team suggest that space agencies and commercial operators consider the recommendations of this report to inform, and mitigate the potential risk posed by VT in future spaceflight.

### Reporting summary

Further information on research design is available in the Nature Research Reporting Summary linked to this article.

## Supplementary information

Reporting Summary Checklist

## Data Availability

No datasets were generated as a result of this research.
